# Advances in Psychiatric Diagnosis: Past, Present, and Future

**DOI:** 10.3390/bs7020027

**Published:** 2017-04-26

**Authors:** Carol S. North, Alina M. Surís

**Affiliations:** 1 Department of Psychiatry, The University of Texas Southwestern Medical Center, Dallas, TX 75390-8828, USA; 2 Metrocare Services, Dallas, TX 75247-4914, USA; 3 VA North Texas Health Care System, 4500 S. Lancaster Road, 151, Dallas, TX 75216, USA; alina.suris@va.gov

**Keywords:** psychiatric diagnosis, nosology, disease classification, biomarkers, DSM-5, controversy, Research Domain Criteria, neuroscience, genetics, medical illness

## Abstract

This editorial examines controversies identified by the articles in this special issue, which explore psychopathology in the broad history of the classification of selected psychiatric disorders and syndromes over time through current American criteria. Psychiatric diagnosis has a long history of scientific investigation and application, with periods of rapid change, instability, and heated controversy associated with it. The articles in this issue examine the history of psychiatric nomenclature and explore current and future directions in psychiatric diagnosis through the various versions of accepted diagnostic criteria and accompanying research literature addressing the criteria. The articles seek to guide readers in appreciating the complexities of psychiatric diagnosis as the field of psychiatry pushes forward toward future advancements in diagnosis. Despite efforts of many scientists to advance a diagnostic classification system that incorporates neuroscience and genetics, it has been argued that it may be premature to attempt to move to a biologically-based classification system, because psychiatric disorders cannot yet be fully distinguished by any specific biological markers. For now, the symptom-based criteria that the field has been using continue to serve many essential purposes, including selection of the most effective treatment, communication about disease with colleagues, education about psychiatric illness, and support for ongoing research.

## 1. Introduction

The articles in this special issue explore psychopathology in the broad history of the classification of psychiatric disorders and syndromes over time as now reflected through the current American criteria, the fifth edition of the Diagnostic and Statistical Manual of Mental Disorders of the American Psychiatric Association (DSM-5) published in 2013. The work in this issue further projects into the future and identifies directions for new developments. Selected topics discussed in this issue include mood disorders, addictions, posttraumatic stress disorder (PTSD), somatoform and dissociative disorders, mild neurocognitive and attention deficit/hyperactivity disorders, catatonia, and homosexuality.

A broad foundation for contemplation of the evolution of diagnostic criteria for specific disorders is provided in the article by Surís, Holliday, and North [1], *The Evolution of the Classification of Psychiatric Disorders*, which traces the history of classification of mental disorders and delves deeply into psychiatric nosology. This article discusses diagnosis in psychiatry as being parallel to that of the larger field of medicine, where the development of systems for classification of medical diseases has been fundamental to the practice of medicine and a cornerstone of medical science. It has long been recognized that diagnosis is key to all medical practice and medical research investigation, a necessary foundation for making treatment decisions, informing prognosis of medical conditions, providing the basis for communication of scientific experts and medical professionals, supporting medical education, determining disease prevalence rates, conducting research, planning for health services and distribution of resources for medical care, and documenting vital public health information [1]. It is still essential for the discipline of psychiatry as a medical specialty to align with medical conventions in categorization of psychiatric illness.

## 2. Controversies

All of the articles in this special issue describe continuing controversies surrounding current criteria. They also review the many changes in the criteria over time. As described by Surís and colleagues [1], the entire diagnostic system of the American Psychiatric Association abruptly became very controversial with the historic release of the third edition of the diagnostic manual (DSM-III). However, the controversy did not end there, with dissatisfaction by growing cadres of professionals arguing for the replacement of the categorical model of psychiatric disease with a dimensional model. More recently, scientists have promoted extensive incorporation of neuroscience and genetics into the definitions of psychiatric disorders [2].

Three main controversies are highlighted in the article on bipolar disorder by Mason, Brown, and Croarkin [3]. One is the concept of mood representing a spectrum of mood states from manic to depressive within the disorder. Another controversy surrounds definition of potential subtypes, especially bipolar II, and relationships of subtypes to the broader category of bipolar disorder. The third major controversy about this disorder is the prevalence of bipolar disorder in children and adolescents as defined by the current criteria.

The article by Robinson and Adinoff on substance use disorders [4] discusses two main current controversies over changes to DSM-5 criteria. First, protracted debates have centered on tensions that have arisen between natural recovery versus disease models, and between abstinence versus harm reduction models. A second controversy surrounds the sparse adoption of evidence-based practices for psychosocial and pharmacological treatments into clinical practice, despite solid evidence for their effectiveness.

In their article on posttraumatic stress disorder (PTSD) criteria in this issue, Pai, Surís, and North [5] examine the controversy surrounding this diagnosis from the time it first appeared in the American diagnostic nomenclature, and before its entry into DSM-III as PTSD [6,7]. Major controversies in the diagnostic criteria for PTSD pertain to the definition of trauma and exposure to it. Almost nothing else about the PTSD criteria has escaped controversy either, including the number, organization, and content of symptom criteria; course definitions; specifiers and subtypes; and special criteria for children under age six in DSM-5.

Few other disorders have garnered as much controversy as the disorders formerly classified as hysteria and related disorders: somatization disorder, conversion disorder, dissociative disorders, and, arguably, borderline personality disorder. As described in the article by North, entitled “The Classification of Hysteria and Related Disorders: Historical and Phenomenological Considerations” [8], disagreement continues to surround the conceptual origins and classification of these disorders. Even the names of these disorders have generated heated debate. The very existence of some of these disorders has apparently been controversial, as illustrated by the disappearance of the longstanding somatization disorder diagnosis and its replacement with somatic symptom disorder in DSM-5.

The article by Carlew and Zartman [9] on neuropsychological disorders focuses especially on attention deficit hyperactivity disorder (ADHD) and mild neurocognitive disorder. The authors noted that the new DSM-5 stipulations for ADHD requiring the presence of symptoms in multiple environments have attracted criticism. The authors also describe controversy over questionable validity of mild neurocognitive disorders in non-geriatric populations and call for additional research to address this new problem in the DSM-5 definition of the disorder.

The article by Wilcox and Duffy [10] addresses a well-established psychiatric syndrome that has never been included as a diagnosis in the DSM system: catatonia. First appreciated as a part of psychotic disorders and then later recognized as more often associated with mood disorders, catatonia has had a lengthy and wandering course in its journey to find its most fitting classification. Wilcox and Duffy succinctly state, “diagnostic parsimony has been long in coming” [10] (p. 577) for catatonia. The authors conclude that this syndrome is caused by a variety of brain diseases.

The article on homosexuality by Drescher [11] describes the most controversial topic of all of the articles in this special issue. What began as a psychiatric disorder in DSM-I and DSM-II, has, with much controversy been overruled with subsequent editions, in response to research and significant discussion as to whether homosexuality constitutes a psychiatric disorder. After this diagnosis was deleted in a subsequent printing of DSM-II in 1973, the diagnosis returned for a short time in DSM-III, where it was limited to ego dystonic cases. Technically, the diagnosis is no longer controversial because it no longer exists in current diagnostic criteria. However, although the diagnosis no longer exists, it is still seen as controversial because therapies designed to treat it continue to garner attention and conflicting opinions.

## 3. Future Directions in Psychiatric Diagnosis

The articles in this special issue illustrate the progress made in diagnostic classification and highlight the controversies that continue to surround efforts to improve and revise diagnostic criteria. DSM-5 continues the tradition of American diagnostic criteria in place since the publication of DSM-III that follows a conceptual paradigm that is empirically-based, atheoretical, and agnostic toward etiology. Today, the psychiatric research field confronts another pivotal point in the conceptualization of psychiatric diagnosis, much as it did in 1980 when DSM-III emerged with monumental changes in the diagnostic system. This time, however, psychiatric research leadership is moving away from the clinically descriptive paradigm that began with DSM-III and seeking a new diagnostic system grounded in neurobiological science. The article by Mason et al. [3] in this special issue describes the proposed NIMH Research Domain Criteria (RDoC) framework and its application to bipolar disorder. Within this framework, biological markers are used to distinguish similar symptoms and symptom clusters across different psychiatric disorders. Resonating with the diagnostic controversies described in the articles in this special issue, the RDoC conceptual framework has generated considerable concerns of its own [3].

## 4. Conclusions

Psychiatric diagnosis has historically followed the lead of medical diagnostic frameworks. Because psychiatric disorders are medical illnesses, it is logical that the same principles of diagnostic classification for other medical disorders should apply to psychiatry. Unlike psychiatric illness, many (but not all) medical diseases have established etiological bases and characteristic biological markers, and thus diagnoses for these medical diseases can be based on biological tests [12], rather than simply based on a characteristic constellation of symptoms—as diagnoses are still defined in psychiatry. A recent movement seeks to incorporate neurobiological elements into the diagnostic criteria for psychiatric disorders [2]. It has been argued, however, that because psychiatric disorders cannot yet be distinguished by any clear and consistent biological markers, it may be premature to attempt to move to a biologically-based classification system for psychiatric diagnosis at this time [13,14,15,16]. The RDoC approach was not intended to supplant current diagnostic systems, and it is not readily conducive to clinical use in classification of psychiatric illness [3,17]. For now, the symptom-based criteria that the field of psychiatry uses continue to serve many purposes, including, as noted in the article by Surís et al. [1], selection of the most effective treatment, communication about disease with colleagues, education about psychiatric illness, and research investigation.
